# Recurrence with pagetoid spread arising 17 years after surgery for intramucosal rectal cancer: a case report

**DOI:** 10.1186/s40792-017-0356-5

**Published:** 2017-07-26

**Authors:** Taichi Matsubara, Yuta Kasagi, Kippei Ogaki, Yu Nakaji, Ryota Nakanishi, Yuichiro Nakashima, Masahiko Sugiyama, Hideto Sonoda, Hiroshi Saeki, Eiji Oki, Yoshihiko Maehara

**Affiliations:** Department of Surgery and Science, Graduate School of Medical Sciences, Kyushu University, Fukuoka, 812-8582 Japan

**Keywords:** Pagetoid spread, Extramammary Paget’s disease, Rectal cancer, Cancer recurrence

## Abstract

**Background:**

Perianal Paget’s disease (pPD) is uncommon, with only about 180 cases documented in the literature. Anorectal carcinoma with pagetoid spread is even rarer.

**Case presentation:**

An 81-year-old woman underwent rectal cancer extirpation with a transanal approach 17 years prior. She has since undergone two reoperations for local rectal cancer recurrence. Then, warts frequently appeared on the vulva on several occasions. Warts appeared on the vulva 1 year ago, which were diagnosed as pPD by biopsy. She underwent perineal tumor resection, and the final histological diagnosis was rectal cancer recurrence with pagetoid spread. The resected stump was positive for cancer cells, and tumor progression was rapid. She underwent additional abdominoperineal resection (Miles’ operation) with lymph node dissection. However, disease progression was rapid and she died 7 months after the Miles’ operation.

**Conclusions:**

There are some case reports describing anorectal carcinoma with pagetoid spread, however, almost of all those cases were synchronous primary anorectal cancer. Here, we report the first case of metachronous recurrence rectal cancer with pagetoid spread arising 17 years after surgery.

## Background

Paget’s disease (PD) is an uncommon intraepidermal adenocarcinoma and is classified as mammary and extramammary. Mammary PD is an adenocarcinoma originating mainly in the mammary duct, and extramammary PD (EPD) is an epidermotropic neoplasm arising from the apocrine glands of organs such as the vulva, penis, scrotum, perineum, and perianal region [1–3]. PD is characterized by the presence of atypical Paget cells with clear cytoplasm and large pleomorphic nuclei in histological diagnosis [4].

Perianal PD (pPD) is a rare disease with only about 180 cases documented in the literature [5]. It is confirmed in approximately 20% of cases of EPD [6], and some studies have shown that pPD may be associated with underlying malignancy [7]. The rate of malignancy associated with pPD ranges from 33 to 86% [8], and it is also associated with underlying anorectal carcinoma [9, 10]. In the case of pPD with underlying malignancy, the carcinoma cells can spread in the anal canal mucosa and perianal skin, and may show histological features similar to Paget cells. Such spread, therefore, is referred to as pagetoid spread [11]. Some case reports describe anorectal carcinoma with pagetoid spread [12, 13]; however, most of those cases were synchronous primary anorectal cancer. To the best of our knowledge, metachronous recurrence rectal cancer with pagetoid spread has never been reported previously.

Thus, we report a rare case of locally recurrent rectal cancer with pagetoid spread arising from intramucosal rectal cancer that was treated by surgery 17 years ago.

## Case presentation

**Fig. 1:**
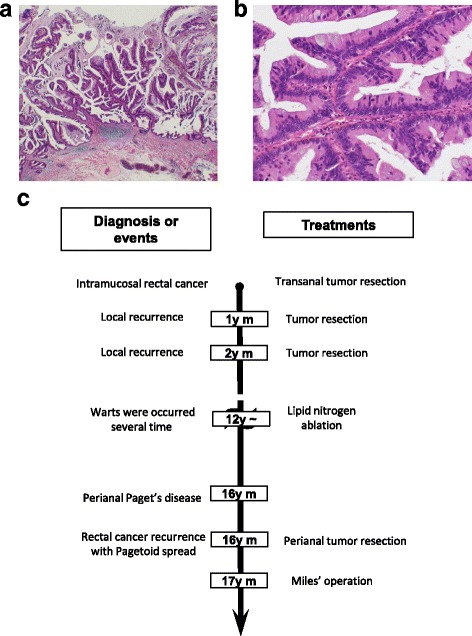
The pathological features of specimen resected after the first operation. The tumor was seen in the intramucosal layer (**a**) (HE, ×20), which was well-differentiated adenocarcinoma (**b**) (HE, ×200). Schematic summary of the patient’s long-term clinical history. *HE* hematoxylin and eosin (**c**)

An 81-year-old woman underwent rectal cancer excision with a transanal approach 17 years ago. The tumor was diagnosed pathologically as intramucosal, well-differentiated rectal adenocarcinoma (Fig. 1a, b). Then, she underwent two reoperations for local rectal cancer recurrence in the next year, and the year after that, she underwent primary resection. Warts frequently appeared on the vulva on several occasions, which were treated each time by liquid nitrogen ablation. The warts also appeared on the vulva 1 year ago. At that time, the tumor was diagnosed as pPD by biopsy, so she underwent perineal tumor resection. The time course of the diagnosis of events and treatment history are shown in Fig. 1c.

**Fig. 2:**
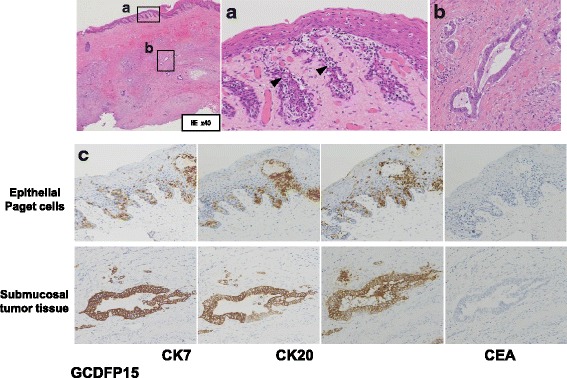
Paget cells were scattered in squamous epithelium (*arrow head*) (**a**) (HE ×200). Adenocarcinoma tissue was observed in the submucosa (**b**) (HE, ×200). Immunohistochemical staining revealed that CK7, CK20, and CEA expression was positive in epithelial Paget cells and submucosal adenocarcinoma tissues. However, GCDFP-15 activity was not detected in any lesions (**c**) (×200)

Pathological diagnosis with hematoxylin and eosin staining revealed that Paget cells were scattered throughout the squamous epithelium, and adenocarcinoma tissue was observed in the submucosa (Fig. 2a, b). Immunohistochemical staining of the tumor showed active immunoreactivity for cytokeratin (CK)7, CK20, and carcinoembryonic antigen (CEA), but the major marker for PD, gross cystic disease fluid protein (GCDFP)-15 was negative (Fig. 2c). Based on these pathological findings, she was diagnosed with intramucosal rectal cancer recurrence with pagetoid spread. Furthermore, the resected stump was positive for cancer cells, and additional resection was considered for her remaining anal lesion.

**Fig. 3:**
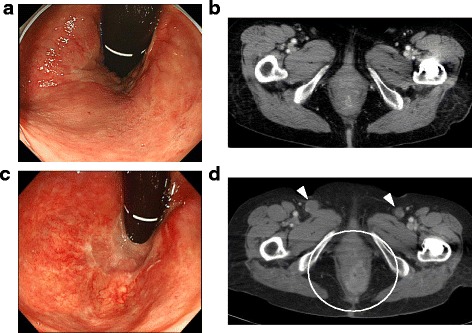
No specific target lesions were observed by rectal endoscopy (**a**) and CT (**b**). Endoscopy showed that anorectal mucous membranes had become rough (**c**). CT revealed thickening of the anorectal wall (circle) and bilateral inguinal lymph node metastasis (arrow head) (**d**)

At the time of her initial visit to our department, she had no particular symptoms, and no specific target lesions were observed by rectal endoscopy and computed tomography (CT) (Fig. 3a, b). Considered she was 81 years old, had long clinical course, surgical invasions, and decline in her quality of life (QOL) if she will underwent radical operations, we decided to follow her progress carefully. Only 5 months later, she experienced severe anal pain. Blood biochemical tests revealed marked increases in tumor makers CEA (171.5 ng/ml) and carbohydrate antigen 19-9 (259.1 U/ml). Endoscopy showed that the anorectal mucous membranes had become rough (Fig. 3c). CT revealed thickening of the anorectal wall and bilateral inguinal lymph node metastasis (Fig. 3d). Because the tumor was growing rapidly and severe anal pain was becoming worse, she underwent abdominoperineal resection (Miles’ operation) with lymph node dissection (perirectal, sigmoid, superior rectal, and lateral without inguinal lymph nodes).

### Pathology

**Fig. 4:**
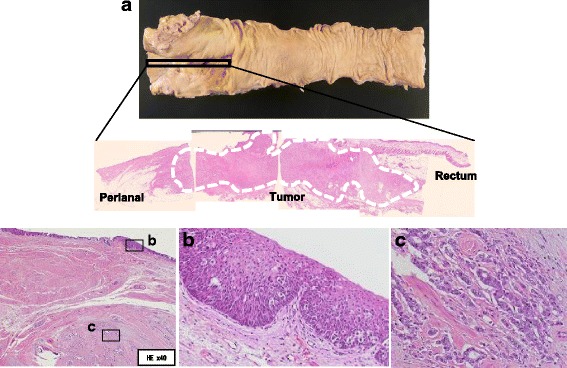
The tumor lesion occupied the perianal region and rectum, with internal anal sphincter invasion (**a**). Many Paget cells (*arrow head*) were observed in the squamous epithelium (**b**). There were moderately to poorly differentiated adenocarcinoma cells in the submucosal layer (**c**)

Pathological examination of the surgical specimen revealed that the tumor lesion occupied the perianal area and rectum, with invasion of the internal anal sphincter (Fig. 4a). Many Paget cells were observed in the squamous epithelium, and there were moderately to poorly differentiated adenocarcinoma cells in the submucosal layer (Fig. 4b, c). The lateral lymph node (no. 283L) was positive for cancer cells. These pathological features were consistent with adenocarcinoma with pagetoid spread.

### Postoperative course

The patient was discharged from hospital on postoperative day 20, and we explained in detail the disease and its histological and clinical conditions to the patient and her family. Because of her age (>80 years) and her performance status 2, neither the patient nor her family wanted any additional therapy. She was transferred to a hospital near her home, and 7 months after the Miles’ operation, she died from multiple metastases of cancer.

## Discussion

The diagnosis of PD must be confirmed histologically. Armitage et al. [14] reported that Paget cells are usually positive for CK7, GCDFP-15, CEA, and human milk fat globule glycoproteins 1 and 2 by immunohistochemical staining. In contrast, pPD associated with rectal adenocarcinoma is usually positive for CK20 but negative for GCDFP-15 [15]. In the present case, histological findings revealed that the resected specimen contained Paget cells in the squamous epithelium as well as adenocarcinoma components in the submucosal layer. Immunohistochemical staining revealed that CK7 and CK20 expression was positive in Paget cells and adenocarcinoma tissues. However, GCDFP-15 activity was not detected in any of the lesions. The patient had a history of intramucosal rectal cancer 17 years ago, and the tumor recurred on several occasions before the present episode. Therefore, we diagnosed the lesion as recurrent rectal cancer with pagetoid spread.

Pagetoid spread is thought to be a type of secondary EPD that can occur with some visceral carcinomas, especially of the rectum and anal canal, leading to epidermotropism with intraepidermal invasion or metastasis. This is known as pagetoid phenomenon [11, 16]. pPD associated with anorectal cancer is also included in secondary EPD, although such cases are rare. Therefore, clinical experience is limited and the literature consists mainly of sporadic case reports and small series. Kubota et al. [17] reviewed 28 cases of pPD associated with anorectal carcinoma, and Goldman et al. [10] reported that pPD with underlying anorectal carcinoma comprises 33% of pPD cases. However, almost all of those reported cases were pPD associated with synchronous primary anorectal cancer. The present case was metachronous rectal cancer recurrence with pagetoid spread with a long-term clinical course after the first operation. This is believed to be the first report to describe such a case.

In the present case, the patient had been diagnosed with intramucosal, well-differentiated adenocarcinoma at the first operation, but final pathological findings revealed that the recurrent tumor was moderately to poorly differentiated adenocarcinoma with pagetoid spread. During perineal tumor resection and Miles’ operation, the tumor exhibited rapid growth and bilateral inguinal lymph node metastases. In general, it is believed that an epigenetic physiological phenomenon is essential for aging and carcinogenesis, and such abnormality mutants are definitive risk factors for cancer [18]. These findings led to speculation that her long-term clinical course, aging, and frequency of resections stimulated changes in cancer epigenetics or tumor characteristics, which might have caused cancer genotype transformation and poor prognosis.

Generally, the prognosis of PD confined to the epidermis is good, however, it is poorer in patients with underlying anorectal carcinoma [19, 20]. pPD associated with malignancy is reported to have a high risk of local recurrence [21]. According to Kubota et al. [17] the 1- and 2-year survival rates of patients with anorectal carcinoma were 91 and 44%, respectively. Armitage et al. [14] indicated the need for multidisciplinary therapy, such as adjuvant chemotherapy and radiotherapy, after the treatment of local recurrence. In our case, the patient and her family did not want any more therapy; therefore, we did not carry out additional chemotherapy and radiotherapy after surgery and the disease took its natural course. Seven months later, she died from multiple metastases of cancer. Our findings suggest that rectal cancer recurrence with pagetoid spread progresses rapidly and has poor prognosis, and it seems to be better to perform additional therapy after surgery.

## Conclusions

In conclusion, this is believed to be the first report of a rare case of locally recurrent rectal cancer with pagetoid spread arising from intramucosal rectal cancer. The present case suggested that radical surgery followed by additional therapies should be considered when recurrence of a rectal cancer with pagetoid spread is diagnosed.

## Abbreviations

CEA: Carcinoembryonic antigen
CK: Cytokeratin
CT: Computed tomography
EPD: Extramammary Paget’s disease
GCDFP: Gross cystic disease fluid protein
PD: Paget’s disease
pPD: Perianal Paget’s disease
