# Correction to: Recurrence with pagetoid spread arising 17 years after surgery for intramucosal rectal cancer: a case report

**DOI:** 10.1186/s40792-018-0415-6

**Published:** 2018-01-25

**Authors:** Taichi Matsubara, Yuta Kasagi, Kippei Ogaki, Yu Nakaji, Ryota Nakanishi, Yuichiro Nakashima, Masahiko Sugiyama, Hideto Sonoda, Hiroshi Saeki, Eiji Oki, Yoshihiko Maehara

**Affiliations:** Department of Surgery and Science, Graduate School of Medical Sciences, Kyushu University, Fukuoka, 812-8582 Japan

## Correction

After publication of the original article [1] the authors noted that the following errors had occurred:

Figure 1c: The vertical line in Figure 1c is misaligned, and partly overlaps ‘12y ~’. The time points in the clinical course are also incorrect and have been updated in this Correction (Revised Fig. 1). In the original article the time points were labelled as 1y m (amended to 1y), 2y m (amended to 2y), 16 y m (amended to 16y 4 m), 16 y m (amended to 16y 7 m) and 17 y m (amended to 17 y 3 m).

**Fig. 1:**
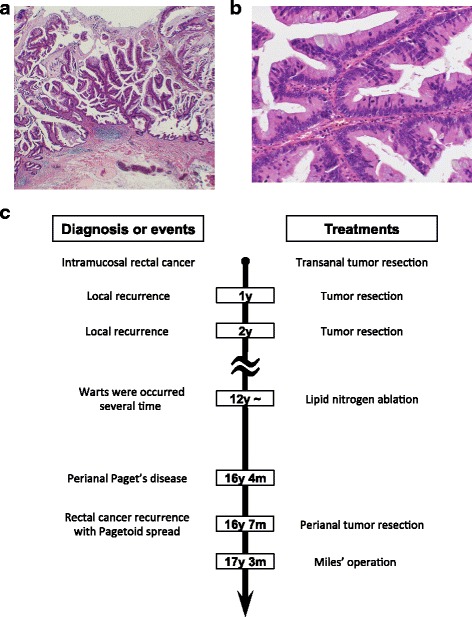
The pathological features of specimen resected after the first operation. The tumor was seen in the intramucosal layer (**a**) (HE, ×20), which was well-differentiated adenocarcinoma (**b**) (HE, ×200). Schematic summary of the patient’s long-term clinical history. HE hematoxylin and eosin (**c**)

Figure 2c: The items for immunostaining (CK7, CK20, CEA, and GCDFP15) are misaligned in Figure 2c and have been updated in this Correction (Revised Fig. 2).

**Fig. 2:**
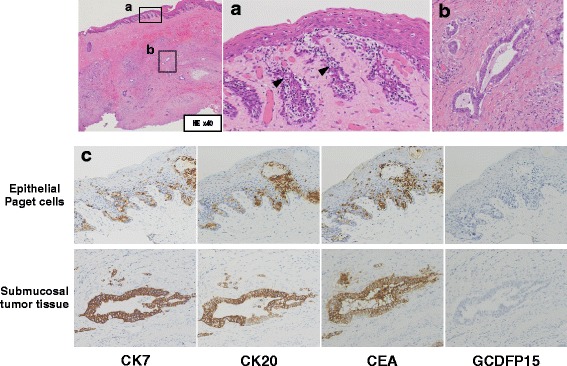
Paget cells were scattered in squamous epithelium (arrow head) (**a**) (HE ×200). Adenocarcinoma tissue was observed in the submucosa (**b**) (HE, ×200). Immunohistochemical staining revealed that CK7, CK20, and CEA expression was positive in epithelial Paget cells and submucosal adenocarcinoma tissues. However, GCDFP-15 activity was not detected in any lesions (**c**) (×200)

The errors do not affect the Conclusions of the original article.
